# Validation of an Improved Statistical Theory for Sea Surface Whitecap Coverage Using Satellite Remote Sensing Data

**DOI:** 10.3390/s18103306

**Published:** 2018-10-01

**Authors:** Haili Wang, Yongzeng Yang, Changming Dong, Tianyun Su, Baonan Sun, Bin Zou

**Affiliations:** 1 School of Marine Sciences, Nanjing University of Information Science & Technology, Nanjing 210044, China; wanghailinew@163.com; 2 Laboratory for Regional Oceanography and Numerical Modeling, Qingdao National Laboratory for Marine Science and Technology, Qingdao 266061, China; yangyz@fio.org.cn; 3 Key Laboratory of Marine Science and Numerical Modeling (MASNUM), First Institute of Oceanography, State Oceanic Administration, Qingdao 266061, China; sunbn@fio.org.cn; 4 Department of Atmospheric and Oceanic Sciences, University of California, Los Angeles, CA 90095, USA; 5 Marine Information and Computing Center, First Institute of Oceanography, State Oceanic Administration, Qingdao 266061, China; sutiany@fio.org.cn; 6 National Satellite Ocean Application Service, State Oceanic Administration, Beijing 100089, China; zoubin@mail.nsoas.org.cn

**Keywords:** whitecap coverage, breaking wave, statistical theoretical model, breaking wave kinetic and potential energy, remote sensing

## Abstract

The whitecap coverage at the sea surface is affected by the ratio of kinetic energy to potential energy, *θ*, the wave spectrum width parameter, ***ρ***, and other factors. This paper validates an improved statistical theory for surface whitecap coverage. Based on the theoretical analysis, we find that the whitecap coverage is more sensitive to ***ρ*** than to *θ*, and the improved statistical theory for surface whitecap coverage is suitable in regions of rough winds and waves. The satellite-derived whitecap coverage data in the westerly wind zone is used to validate the improved theory. The comparison between the results from theory and observations displays a better performance from the improved theory relative to the other methods tested.

## 1. Introduction

Breaking wave processes are of importance to the air–sea interaction, coastal circulation, ocean remote sensing and offshore engineering. Wave breaking can be a good visual indicator of wave–current interactions [1], where the upper-ocean currents and mixing are partially driven by the wave breaking [2]. However, previous studies still have not been able to establish a comprehensive understanding of wave breaking in deep ocean. Zhang et al. [3] use a vertical distribution model of turbulent kinetic energy based on an exponential distribution method, and they demonstrate that the energy dissipation rate of breaking waves relies on wind speed and the sea state. He and Song [4] examine the onset and the strength of unforced wave breaking in a numerical wave tank, and they suggest that the application of energy growth rate can yield better results than using the initial wave steepness for estimating the fractional energy losses.

In the study of wave breaking characteristics, the whitecap is one of the most important phenomena for wave breaking. Whitecaps are the manifestation of the breaking wave on the surface. As elucidated in literature, whitecaps correlate strongly with the energy dissipated through wave breaking [5,6,7,8,9]. A range of about 30–50% of wave energy is required to generate a bubble cloud [10,11] which can overcome buoyancy and invade the water body. Zhao [12] indicates that sea spray resulting from wave breaking can be distinguished as a film, a jet, or spume droplets. Monahan [13] points out that the total fraction of the whitecap includes two stages, A and B. Stage A—dynamic foam patches of the initial breaking; Stage B—static foam patches during the whitecap decay, which consists of “fossil foam” or “foam rafts”, fossil foam is the foam left over the surface of the previous breaking that occurs two or more times, and foam rafts are floating bubble rafts [14]. Potter et al. [15] use the brightness temperature at the horizontal and vertical polarizations to distinguish active and residual whitecaps. A novel method to measure the fractional coverage, intensity, and decay time of whitecaps using radiometry above the water is developed by Randolph et al. [16], using data collected in the Southern Ocean. Callaghan et al. [17] report on the measurements of *W* made in the North Atlantic as part of the Marine Aerosol Production (MAP) campaign from the summer of 2006, and they propose the relationship of oceanic whitecap coverage to wind speed and wind history, and at above 9.25 m/s, whitecap coverage is generally found to be larger in periods of decreasing wind speeds than in periods of increasing wind speeds. Dyachenko and Newell [18] detail the processes of whitecaps in areas where the wind is strong and the sea surface is dominated by sharp crested waves.

Although only a few percent of the sea surface is covered by whitecaps, they have a significant influence on the brightness temperature of the sea surface, as seen by passive radiometer instruments [6,19,20], and they also affects satellite remote sensing of the ocean color [21,22]. Accurate estimates of the whitecap coverage on the surface due to wave breaking is a vital requirement for improving upper-ocean turbulence models, wave models, and for research in ocean–atmosphere interactions. Whitecaps also play a significant role in the transfer of gases at the air–sea interface [23,24,25,26]. Therefore, understanding the process involving whitecaps can improve the calculation of gas fluxes between the ocean and the atmosphere [27].

An increasing number of researchers are focused on developing innovative methods to accurately parameterize the whitecap coverage for the modeling of whitecap-dependent processes. Almost all current parameterizations of the whitecap coverage are defined as a function of wind speed. However, there is substantial evidence that the whitecap coverage is also influenced by the wave field and other environment conditions [20]. Romero et al. [1] show that the measured whitecap coverage well correlates with the spectral moments for wavenumbers that are larger than the spectral peak. To accurately determine the whitecap coverage, Guan and Sun [28], and Guan et al. [29] improve the analytical whitecap model of Kraan et al. [7] by estimating the undetermined constant in the model. Yuan et al. [30] present the equations of the statistical theory of breaking entrainment depth, and the whitecap coverage of real sea waves, which are directly related to the wave characteristics, where the fraction of the whitecap coverage includes stages A and B. By applying an intensity threshold method, Anguelova and Hwang [31] propose an approach to obtaining an active whitecap fraction using energy dissipation rate data to extract the fraction of the active whitecap coverage from photographs. Wang et al. [32] improve the statistical theoretical model for wave breaking based on the ratio of breaking wave kinetic energy to potential energy. Their results are demonstrated to be more accurate than that of Yuan et al. [30]. Validation of the improved theoretical model for the general sea state has not been done by Wang et al. [32], due to limited observed data in the open ocean. With progress in satellite remote sensing technology, it is now possible to obtain reliable data for the validation of the improved statistical theoretical model. Such a validation is crucial for studying whitecap-dependent processes.

This paper focuses on testing the statistical theory for whitecap coverage for the general wave state [30] using improved parameters from Wang et al. [32]. The remainder of this paper is organized as follows. Section 2 describes the whitecap coverage model and introduces the empirical equations versus the wind velocity relationship. Section 3 presents the model analysis, testing of the ratio of the breaking kinetic energy to potential energy, and the comparison of the whitecap coverage model with satellite data. Section 4 gives the conclusions of the study.

## 2. Model Description

### 2.1. Parametric Expressions of Whitecap Coverage

**Figure 1:**
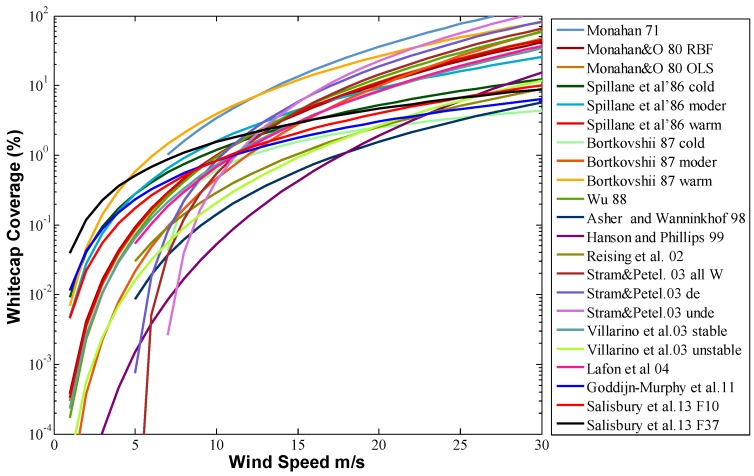
Wind speed empirical relationships. See Table 1 for details.

The whitecap is usually quantified as the fraction of the whitecap coverage, *W*, on the ocean surface. Most parameterizations are a function of wind speed. In past studies, various empirical equations versus wind velocity have been derived. Table 1 lists several of the equations, and Figure 1 shows the semi-log plot of the whitecap. *W* is the whitecap coverage. $U_{10}$ is the wind speed at 10 m above the sea surface. All curves conform to the envelope curve, which was first proposed by Monahan [33].

W increases with wave age [44] and wave height [27,45]. Cross-swell conditions, on the other hand, reduce whitecapping [43]. Considering many factors, the expression of whitecap coverage, given in Yuan et al. [30], for a sea state with infinite wind fetch and duration, the ratio of the breaking kinetic energy to potential energy θ is analyzed in Wang et al. [32], and the values $\theta =8,\;C_{en}=0.1777,\;n=-1.713,\;F_{T}=0.75$ are obtained for a sea state with infinite wind fetch and duration; $C_{en}$ and *n* are constants. Parameter $F_{T}$ is a dimensionless number that represents the integral of the bubble accumulation function over the entire domain (the value of $F_{T}$ is within the interval 0–1) [30]. We also consider similar values to test the theoretical expressions of the whitecap coverage for the general sea state.

### 2.2. Theoretical Expressions of the Whitecap Coverage for the General Sea State

The expression of the whitecap coverage for the general sea state is given in Yuan et al. [30] as follows:

(1) W=FTUBρ4απ(gL¯λπ)12Cen[(1+θ)α2π2λ24ρ2(HSL¯)2]n×exp{−ρ22α2π2λ2(HSL¯)−2ϕ02}

where:

(2) ϕ02=[1−0.55×(2απλCD)121ρ(U102gL¯)12]4

$U_{B}\approx 0.25\;m\;s^{-1}$ is the minimum terminal rise speed for the bubble group concerned [30,32]. *g* is the acceleration due to gravity, ρ is a parameter associated with the spectrum width (more details are given in Section 3.2), α=1 for weak nonlinear waves, π=3.14 [30]. $\bar{L}$ is the mean wavelength, and λ=2/3 is the coefficient derived from the Neumann spectrum, which is comparable to 0.87 as measured in the laboratory [30,32,46]. $H_{S}$ is the significant wave height. $\bar{L}=g\lambda T_{z}^{2}/2\pi$, $T_{z}$ is the zero-crossing wave period [30,32], $C_{D}=1.5\times 10^{-3}$ is the drag coefficient [47].

Applying the above equations to the general sea state and substituting the values of $C_{en},\;n,\;F_{T}$ [32] into Equation (1), *W* can be re-written as:

(3) W=3ρ4π(3gL¯2π)12×0.1777[(1+θ)π29ρ2(HSL¯)2]−1.713×exp{−9ρ28π2(HSL¯)−2ϕ02}

where the significant wave height and the zero-crossing wave period $T_{z}$ are derived using the third Marine Science and Numerical Modeling (MASNUM) wave model [48,49,50,51], $T_{z}$ is used to calculate the wavelength, $\bar{L}$, and the whitecap coverage (Equation (3)) is directly obtained from $H_{S}$ and $\bar{L}$.

The third MASNUM wave model is developed by Key Lab of Marine Science and Numerical Modeling, State Oceanic Administration, China. In the model, the wave energy spectrum balance equation and its complicated characteristic equations are derived in wave-number space. The characteristic inlaid method is applied to integrate the wave energy spectrum balance equation [48,49,50,51]. MASNUM is widely used in forecasting ocean wave movement [51]. The MASNUM wave model shows good results in the general sea state and good improvement in the high sea state, and the model is used to evaluate the wave-induced mixing in the upper ocean [48,49,50,51].

## 3. Validation

### 3.1. Data

The model results of the whitecap in the general wave state are compared with observed data are reported. The observed whitecap is taken from Salisbury et al. [20], which we digitalize for the month of October 2006 and then extract the data points.

The method of estimating the whitecap coverage from satellite remote sensing data is described in Anguelova and Webster [19]. Salisbury et al. [20] refer to this method as the $W(T_{B})$ algorithm. The $W(T_{B})$ algorithm has been improved in several aspects [52,53], such as the use of independent input datasets in the algorithm. Salisbury et al. [20] attest the variability of the whitecap coverage extracted by the $W(T_{B})$ algorithm. The algorithm for estimating W combines the satellite *T_B_* observations with models for the rough sea surface and the foam-covered areas (whitecaps). An atmospheric model is used to obtain the changes in *T_B_* at the ocean surface. Wind speed, wind direction, the sea surface temperature, and atmospheric variables such as water vapor and cloud liquid water are necessary as inputs to the atmospheric, roughness, and foam models; for simplicity the algorithm is called the *W*(*T_B_*) algorithm [20]. The datasets used are shown in Table 2. The resolution of the whitecap coverage data is 0.5° × 0.5°.

We extracted the data points of the satellite-derived *W* estimates in Salisbury et al. [20] for the month of October 2006, to validate the improved equation for the surface whitecap coverage. We extracted 354,521 data points in the latitude range of 90° S to 89° N and the longitude range 180° E to 180° W. We then mapped these data onto 0.5° × 0.5° resolution grids. The wave parameters $H_{S}$ and $T_{z}$ in Equation (3) are calculated using the third MASNUM Wave Model [48,49,50,51]. QuikSCAT wind data [20] has 0.5° × 0.5° resolution and is applied to the third MASNUM Wave Model.

### 3.2. θ and ρ

*θ*, first introduced by Yuan et al. [30], is the ratio of the breaking wave kinetic energy to the potential energy. The wave-breaking process mainly occurs near the crest of the wave front. Based on the definition by Yuan et al. [30], *θ* is further simplified by Wang et al. [32], and the values 8–11 lie in the middle of *θ*’s distribution, so that in this study, *θ* was kept between 8 and 11 for the general state of the ocean.

$\rho^{2}=\frac{\mu_{2}^{2}}{\mu_{0}\mu_{4}}$, $\varepsilon^{2}=1-\rho^{2}$, ε is the spectrum width parameter, $\mu_{i}$ is the *i*th-order moment of the wave frequency spectrum, and *ρ* is a parameter associated with the spectrum width [30]. Since the Neumann spectrum describes the well-developed wave field [46]. Yuan et al. [30] apply the spectrum to derive the practical expressions of the whitecap coverage for a sea state with infinite wind fetch and duration. The spectral moment ($\mu_{0}$, $\mu_{2}$, $\mu_{4}$) can be obtained through the definition of the spectral moment [30], such that $\rho^{2}=\frac{\mu_{2}^{2}}{\mu_{0}\mu_{4}}=\frac{1}{3}$, $\varepsilon^{2}=\frac{2}{3}$ for a sea state with infinite wind fetch and duration, in Yuan et al. *ρ*^2^ = 1/3, so here we choose 0.53–0.59 for a test, and the values of the spectral width ε are from 0.80 to 0.84, which represent the general sea state.

**Figure 2:**
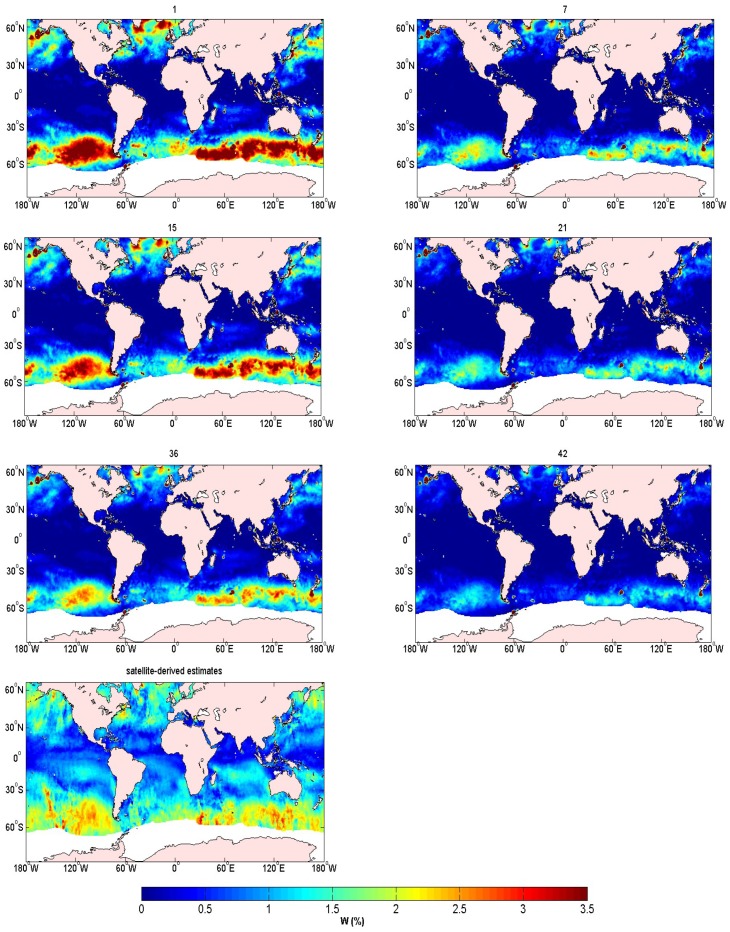
Global whitecap coverage in October 2006 for combination numbers 1, 7, 15, 21, 36, and 42 (See Table 2 for more details about the different combinations) and the satellite-derived estimates of the global whitecap coverage in October 2006. A unified scale colorbar with values from 0 to 3.5 % is used.

To explore the dependence of whitecap coverage on *θ* and *ρ*, different combinations are chosen (Table 3), where *θ* is kept between 8 and 11, and *ρ* varies from 0.53 to 0.59, with intervals of 0.6 and 0.01, respectively. The numbers in Table 3 from 1 to 42 refer to the different combinations of *θ* and *ρ*. For example, number 1 represents the combination of *θ* = 8 and *ρ* = 0.53. We substituted different values of *θ* and *ρ* into Equation (3), and obtained 42 results of whitecap coverage under different combinations of *θ* and *ρ*. Several results are shown in Figure 2. Each figure exhibited patches of high and low values over the study area. Whitecaps appeared in regions with wind exceeding approximately 3 m s^−1^ [33,40]. The value of the whitecap coverage obtained near the equator was very small, and several large value centers were observed in the westerly zone in the Southern Hemisphere. Figure 2 (1, 15, 36) and Figure 2 (7, 21, 42) show that the whitecap coverage decreased with increasing *θ*. Figure 2 (1, 7), Figure 2 (15, 21), and Figure 2 (36, 42) show that the whitecap coverage decreased with increasing *ρ*. The effect of *ρ* on the whitecap coverage was greater than that of *θ* on the whitecap coverage, as Figure 2 shows that the change in the whitecap coverage value is larger with the variation of *ρ* than that of *θ*. To determine how *θ* and *ρ* accounts for the variability in the whitecap coverage as estimated by the model, we calculated the Pearson product-moment correlation coefficient (*r*) between the satellite measurements and the model results with different combinations of *θ* and *ρ*. The correlation coefficient was calculated using the following formula:

(4) r=(∑XY−∑X∑YN)/(∑X2−(∑X)2N)(∑Y2−(∑Y)2N)

where *X* is the model whitecap coverage, and *Y* is the satellite whitecap coverage. The number of the data N is 128,944. There are 128944 satellite-derived data and 128944 model data used, within a latitude range of 79° S to 65.5° N and a longitude range of 180° E to 180° W. The corresponding correlation coefficients are shown in Table 4. It was shown that the correlation between the satellite measurements and the model results decreased with increasing *ρ*, but the correlation coefficient varied within a small range and irregularly with a change in *θ*. Therefore, Figure 2 and Table 4 show the result: the whitecap coverage was more sensitive to *ρ* than *θ*.

**Figure 3:**
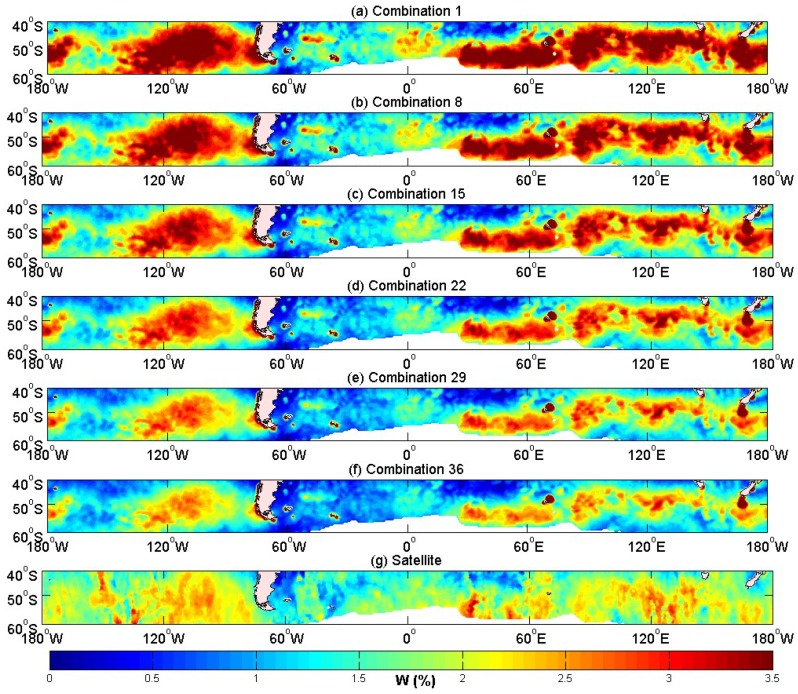
The whitecap coverage of the Equation (3) in October 2006 over 180° W–180° W,40° S–60° S for (**a**) Combination 1, *θ* = 8, *ρ* = 0.53; (**b**) Combination 8, *θ* = 8.6, *ρ* = 0.53; (**c**) Combination 15, *θ* = 9.2, *ρ* = 0.53; (**d**) Combination 22, *θ* = 9.8, *ρ* = 0.53; (**e**) Combination 29, *θ* = 10.4, *ρ* = 0.53; (**f**) Combination 36, *θ* = 11, *ρ* = 0.53; (**g**) satellite-derived result [20]. A unified scale colorbar with values from 0 to 3.5 % is used.

According to Table 4, several combinations with higher correlation are selected as representatives to further validate the model. One can clearly see that the combinations 1, 8, 15, 29 and 36 (in Table 3) had higher correlations than other combinations. A significant occurrence of whitecap coverage was presented in the westerly region (180° W–180° W, 40° S–60° S). This area was therefore chosen for further validation of the whitecap coverage model (Figure 3). Figure 3a–f shows that the whitecap coverage decreased with increasing *θ* when *ρ* was kept constant. Several large value centers were observed in the westerly zone, with the distribution of the centers resembling that from the satellite-derived results. However, the values of the whitecap coverage obtained in the coastal areas were noted to be larger than in the satellite observations. For example, the whitecap coverage in the coastal areas around 70° W, 70° E and 170° E were not well reproduced by the model. By comparing color scales, where the color represents the value of the whitecap coverage, the value of the whitecap coverage in Figure 3a–f was decreasing. The value of the whitecap coverage of Figure 3f was the closest to Figure 3g, and the distribution of the center of the whitecap coverage of Figure 3f was similar to Figure 3g. Figure 3 shows that the performance of Combination 36 was the best. *θ* = 11 and *ρ* = 0.53 for Combination 36 were thus chosen in the following validation and substituted into Equation (3).

**Figure 4:**
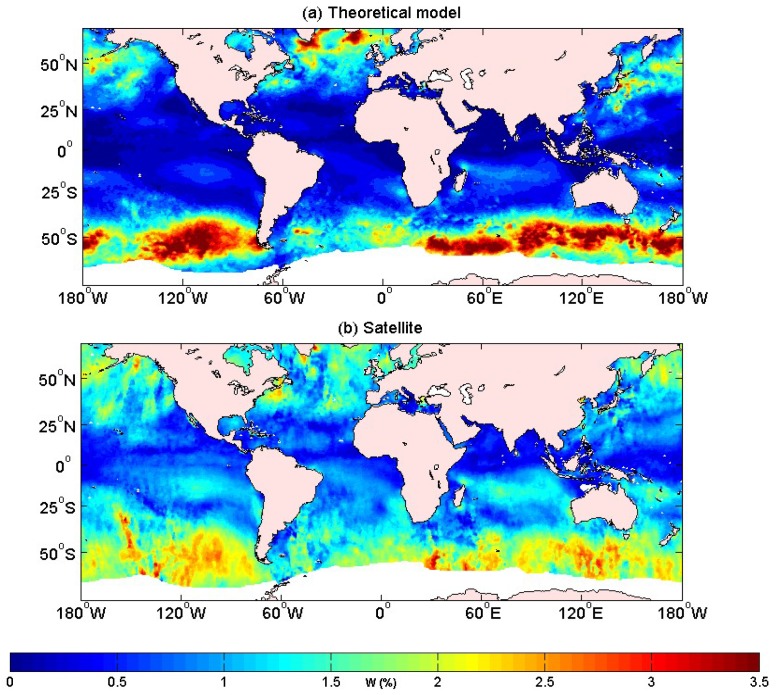
The whitecap coverage in October 2006 over 180° W–180° W,55° S–40° N, for (**a**) model results using Combination 36 (*θ* = 11, *ρ* = 0.53) in Equation (3), and (**b**) satellite-derived results [20].

In the following model analysis, we analyzed the errors of the model data with the satellite data. We analyzed the distribution of the global whitecap coverage over 180° W (E)–180° W, 55° S–40° N. We then carried out a regional analysis. Figure 4 displays that both the whitecap coverage of the model and the satellite had extreme centers of high values, and the spatial distribution was very similar, but there was still some difference. The picture shows that our theoretical model underestimated the breaking processes in the equatorial area. The whitecap coverage of the theoretical model due to the weaker wind near the equator, the breaking process was not as significant as in the westerly areas. Our theoretical model thus required further improvement, especially in the areas with weak winds, like the equatorial area.

### 3.3. Model Analysis

**Figure 5:**
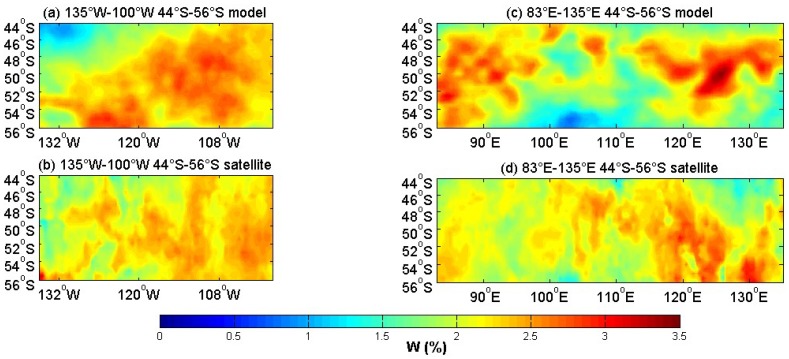
The whitecap coverage over the first part (44° S–56° S, 135° W–100° W) from (**a**) the model and (**b**) the satellite, and over the second part (44° S–56° S, 83° E–135° E) from (**c**) the model and (**d**) the satellite.

**Figure 6:**
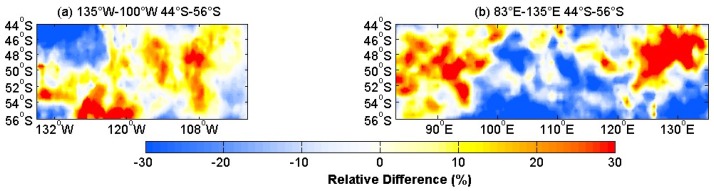
(**a**) The relative differences of the whitecap coverage over the western part (44° S–56° S, 135° W–100° W). $RD=\left(W_{\mathrm{mod}}-W_{\mathrm{obs}}\right)/W_{obs}\times 100$, RD is the relative difference, $W_{\mathrm{mod}}$ is the whitecap coverage of the model, $W_{obs}$ is the whitecap coverage of the satellite-derived data, and (**b**) is the relative differences over the east part (44° S–56° S, 83° E–135° E).

**Figure 7:**
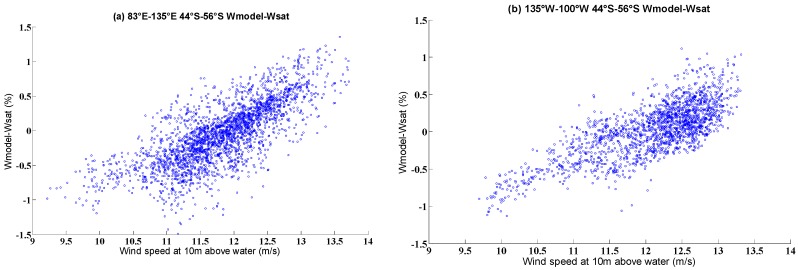
Scatter distribution of the comparison of errors (model–satellite) between our whitecap model and satellite data, over (**a**) 44° S–56° S, 135° W–100° W and (**b**) 44° S–56° S, 83° E–135° E.

Figure 5 shows the theoretical model result and the satellite data. The model outputs were compared to satellite measurements over 135° W–100° W, 44° S–56° S and 83° E–135° E, 44° S–56° S. These areas were in the westerly belt, and they were mainly areas of large waves with a significant breaking process. The area spanning 135° W–100° W and 44° S–56° S, and 83° E–135° E and 44° S–56° S were referred to as the western part and eastern part, respectively. The picture displays in Figure 5a–d had extreme centers of high values, and the spatial distribution of (a) and (b) were similar, and the spatial distribution of (c) and (d) was also similar, but there was still some difference. The relative differences of the whitecap coverage in Figure 6 showed more information. Figure 6 shows the centers of the positive and negative anomalies. The positive anomalies corresponded to the highest whitecap coverage, which meant that the model overestimated the whitecap coverage. This may be due to the wind speed. More studies are shown in Figure 7.

The maximum and mean values of the whitecap coverage over the western and eastern parts were compared when *θ* = 11 and *ρ* = 0.53. The comparison results are shown in Table 5 and Table 6. For the western part, the percentage difference (|model result−satellite result|/satellite result × 100) between the model and satellite mean maximum values was 6.3%, and the percentage difference between the model and satellite mean values was 1.2%. For the eastern part, the percentage difference between the model and satellite maximum values was 11.4%, and the percentage difference between the model and the satellite mean values was 2.8%. The percentage difference of the mean value was small; the mean values of the satellite-derived and modeled whitecap coverage are similar. These results demonstrate that whitecap coverage can be well reproduced in these areas. However, there were deviations due to factors such as sea state and the atmospheric impact. Figure 7 displays the dependence of the whitecap coverage model error on wind. The Figure 7 shows that most of the error data was concentrated between −0.5% ~+0.5%. When the wind speed was lower than 10.8 m/s, the deviation was negative. When the wind speed was between 10.8 and 12.9 m/s, the deviation had both negative and positive values. When the wind speed was greater than 12.9 m/s, the deviation was positive. Such variations resulted from the influence of the wind speed, wind history, and the measurement of both stage A and stage B whitecaps [16]. As wave parameters ($H_{S}$,$T_{z}$) were included in our model, the whitecap coverage of the model result was affected by the wind speed and the wave age. Nordberg et al. [56] and Ross and Cardone [57] point out that the presence of foam streaks above about 13 m/s and the ratio of the area of foam streaks to whitecaps increased with increasing wind speed, which may be a reason explaining the positive deviations for wind speeds higher than 12.9 m/s. Meanwhile, the wind speed could affect the detection of the whitecaps by the satellites.

Through the least squares method, Zhao et al. [45] make a regression analysis of the whitecap coverage as a function of wind speed, wave age, wave period, friction velocity, and breaking parameter $R_{B}$ respectively. It also demonstrates a strong dependence of the whitecap coverage on the wave parameters. The correlation coefficient of $R_{B}$ is the highest. Thus, the following Equation (5) was chosen for testing:

(5) $$W=3.88\times 10^{-5}R_{B}^{1.09},$$

**Figure 8:**
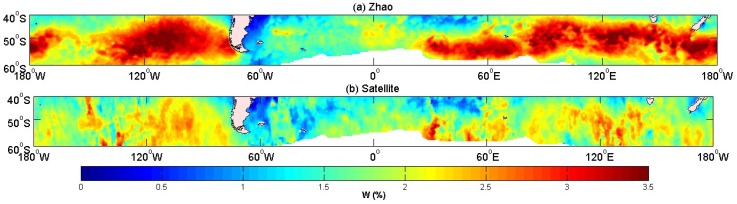
Distribution of the whitecap coverage over 40° S–60° S and 180° E–180° W. (**a**) The results of Zhao’s whitecap coverage relationship [45], and (**b**) satellite-derived results [20].

$R_{B}=u_{*}^{2}/(\upsilon \omega_{p}),\;\omega_{p}=2\pi /T_{P},\;u_{*}^{2}=C_{D}U_{10}^{2},\;\upsilon =0.15\times 10^{-4}\left(m^{2}/s\right)$, $u_{*}$ is the friction velocity, $\omega_{p}$ is the peak angular frequency of wind-waves, $T_{P}$ is the wave period, and υ is the kinematic viscosity of water. Here, we chose the largest value $\upsilon =0.15\times 10^{-4}\left(m^{2}/s\right)$ in Zhao et al. [45] (if it was set to be smaller, the value of the whitecap coverage would be too greatly overestimated). The result is shown in Figure 8. The plot showed that the value of the whitecap coverage was overestimated compared with the result of the satellite-derived coverage. There are very large areas, where (a) shows whitecap coverage, but none are observed in (b). Thus, the relationship between the whitecap coverage and the breaking parameter still needs further improvement.

**Figure 9:**
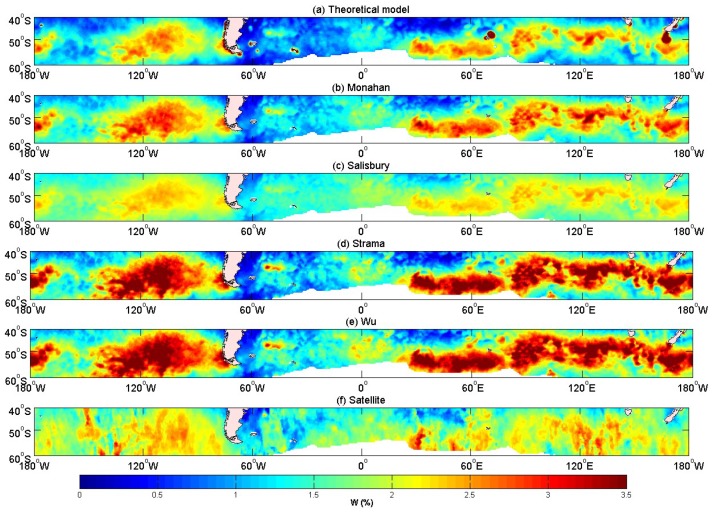
Distributions of the whitecap coverage from different models over 40° S–60° S and 180° E–180° W. (From top to bottom): (**a**) Statistical theoretical model of the whitecap coverage ($C_{en}$ = 0.1777, *n* = −1.713, θ = 11, $F_{T}$ = 0.75, *ρ* = 0.53), (**b**) Monahan’s empirical relationship [58], (**c**) Salisbury’s fitting formula [20], (**d**) Strama’s empirical relationship [40], (**e**) Wu’s empirical relationship [37], and (**f**) satellite-derived result [20]. A unified scale colorbar with values from 0 to 3.5% is used.

According to the results above, our model domain was set between 40° S–60° S and 180° E–180° W. The model results were then compared with satellite measurements from Salisbury et al. [20], and with several widely accepted wind speed empirical relationships (Table 7). We used Equation (3) to calculate the fraction of the whitecap coverage. The results of the whitecap coverage mean values in October 2006 are shown in Figure 9.

All of the models displayed large value centers in the westerly zone. Additionally, some models showed overestimated values at and around the large value centers in Figure 9. Since there was almost no land obstruction in the Southern Hemisphere (40° S–60° S), the sea water moved toward the east from the west under the influence of wind where the sea area was affected by the westerly wind all year round. The winds and waves in this area were high, resulting in a generally large the whitecap coverage. Scanlon et al. [59] indicate that the dissipation source term is more closely related to the whitecap coverage. In Yuan’s model [30], the whitecap coverage is not only dependent on the wind speed and the distribution of the wind, but also on the energy dissipation and the other wave parameters, especially the wave steepness. The top panel in Figure 9, which was the result from our improved model, shows that near the land, the values were overestimated, suggesting that our model was again not suitable in coastal areas. This may be due to errors in calculating wave steepness or uncertainties associated with the energy dissipation rate. The whitecap coverage of our model was based on the ratio of the breaking wave kinetic energy to the potential energy. In coastal areas, the whitecap coverage was also much influenced by the topography. However, the model performed well in the open ocean, with values ranging from 0 to 3.5%. The value of the whitecap coverage was almost similar to that of the satellite-derived result. The spatial distribution of the whitecap coverage was better than Figure 8a. Based on Monahan’s empirical relationship, *W* = 4.5 × 10^−6^ *U*^3.31^, the value of the region around the maximum value center was more than 3.5% in the westerly areas. The distribution was noted to have similar pattern with the wind field (QuickSCAT), suggesting a high dependence on the wind field. Figure 9d,e has the same characteristics, and the value of the whitecap coverage was larger than that in Figure 9b. Figure 9c is parameterized as a function of wind speed by fitting power laws [20]. The values also exhibited a dependence on the wind.

**Figure 10:**
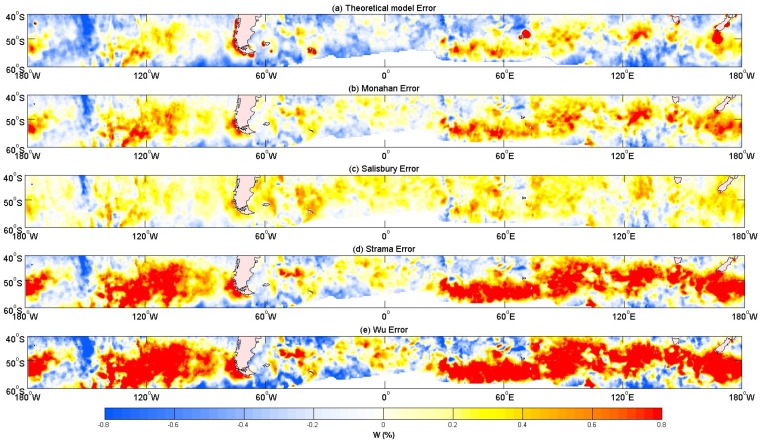
Comparison between errors from different whitecap models, and satellite data over 40° S–60° S and 180° E–180° W (from top to bottom): (**a**) statistical theoretical model of the whitecap coverage ($C_{en}$ = 0.1777, *n* = −1.713, θ = 11, $F_{T}$ = 0.75, *ρ* = 0.53), (**b**) Monahan’s empirical relationship [58], (**c**) Salisbury’s fitting formula [20], (**d**) Strama’s empirical relationship [40], and (**e**) Wu’s empirical relationship [37]. A unified scale colorbar with values from −0.8 to 0.8% is used.

Figure 10 shows the error, which is calculated as the model results minus satellite results. The fraction of the whitecap coverage based on the wind speed empirical relation was dependent on the wind. Compared to the fraction of the whitecap coverage derived from the satellite data, a big difference in the value and spatial distribution was observed. Most in situ datasets of the whitecap coverage have been obtained in coastal, fetch-limited conditions [19]; data from the open ocean are very sparse [20]. The empirical formulas of the whitecap coverage listed in Table 1 were developed from the situ data and they were not applicable to the open ocean. The reason is that the whitecap coverage is affected by many factors: sea conditions, breaking parameters and atmospheric factors, but the above empirical formulas in Table 7 only included the wind speed (Table 7 shows the most commonly used formulas among all those listed in Table 1), and they were very dependent on the wind field. Further, Figure 10 shows that the present model (Figure 10a) well captured the fraction of the whitecap coverage, as compared to those from empirical formulas, in terms of the value and spatial distribution in the westerly zone, and the error of the present model was smaller than those from the empirical formulas. Therefore, the present model is applicable to the open ocean. It is also noted that the present model generated large errors, and the whitecap coverage is overestimated in the coastal areas. The large error may be caused by several factors, such as errors in the satellite-derived whitecap coverage, errors introduced when extracting data from a digitalized image, and errors from the present model, which are based on the ratio of the breaking wave kinetic energy to the potential energy, and wave breaking in the coastal areas are more complicated.

## 4. Discussion and Conclusions

### 4.1. Discussion

Whitecaps are usually the “tuning knob” of a wave model [8]. The fraction of the whitecap coverage can be used to improve upper-ocean turbulence models, wave models, and can be used for research in ocean–atmosphere interactions. An accurate estimation of whitecap coverage has significant practical applications, such as in ocean observation and modeling. In this study, a more accurate surface whitecap coverage is estimated, based on the improved statistical theoretical whitecap coverage model.

Wang et al. [32] have improved the constants of the whitecap model from Yuan et al. [30]. In this paper, the improved whitecap model is used to calculate the whitecap coverage, and the results are compared to those from Salisbury et al. [20]. For the whitecap model [30], *θ* and *ρ* vary within a range of values, in order to investigate the sensitivity of whitecap coverage *W* to the parameters. *θ* and *ρ*, which are negatively correlated to whitecap coverage, are demonstrated to have similar effects on the value of the whitecap coverage. Table 4 shows that the correlation between the satellite and model results decrease with increasing *ρ*, but they cannot determine the relationship with *θ*. As the correlation coefficient of the whitecap coverage is more sensitive to *ρ* than *θ*, the whitecap coverage is more dependent on *ρ* than *θ*. Many factors affect the whitecap coverage, therefore requiring more improvement to the statistical theoretical whitecap model. Table 5 and Table 6 show that the maximum and mean values are almost the same as the satellite data. There will be deviations due to factors such as sea state and the atmospheric impact. Figure 9 and Figure 10 show the poor performance of the whitecap model in coastal areas, while good results are observed in the open ocean. Values near islands are noted to be overestimated by the model.

The present model produces better results than the above wind speed-related empirical formulas. Analyzing the whitecap model result, the formula for surface whitecap coverage is more suitable in high winds and waves areas, while the values of the whitecap coverage obtained near the equator and middle latitude are not well simulated (the value is underestimated). This is because the whitecap coverage of our model (Equation (3)) was developed based on the ratio of the breaking wave kinetic energy to potential energy, and it also includes the wind speed and wave parameters. It can cover the wave breaking condition in the open ocean, but it does not capture the conditions in coastal areas well, where the wave breaking conditions are more complicated, such as bottom topography effects.

### 4.2. Conclusions

Our whitecap coverage model still needs more improvement. The model is more suitable for the large wave areas in the open ocean, but in tropical areas and middle latitudes, the ratio of breaking wave kinetic energy to potential energy is set to be constant, which should be improved further. There are still other reasons leading to the underestimate results of the whitecap coverage. The model can further be improved, especially in the equatorial zone and in the coastal areas. Other dynamic factors also need to be considered in order to determine the exact coefficient and parameters for the statistical theoretical model. Different measurements of the degree of wave age and mean wave slope can account for 80 to 85% of the variability in whitecap coverage [20]. The minimal wind speed for wave breaking, bubble persistence time, and the wave number spectrum also influence the whitecap coverage. Further work is still required to provide a greater insight into the environmental and meteorological factors affecting whitecap formation on the sea surface.

## Figures and Tables

**Table 1:** Statistics on the relationship between whitecap coverage and wind speed, *W* is the whitecap coverage. $U_{10}$ is the wind speed at 10 m above the sea surface.

No.	Reference	As Referred to in Figure 1	Equation	Note
1	Monahan [33]	Monahan 71	$W=1.35\times 10^{-3}U_{10}^{3.4}$	As a percentage, U > 7 m/s
2	Monahan and O’Muircheartaigh [34]	Monahan & O 80 RBF	$W=3.84\times 10^{-6}U_{10}^{3.41}$	
3	Monahan and O’Muircheartaigh [34]	Monahan & O 80 OLS	$W=2.95\times 10^{-6}U_{10}^{3.52}$	
4	Spillane et al. [35]	Spillane et al.’ 86 cold	$W=9.279\times 10^{-5}U_{10}^{2.112}$	
5	Spillane et al. [35]	Spillane et al.’ 86 moder	$W=4.755\times 10^{-5}U_{10}^{2.525}$	
6	Spillane et al. [35]	Spillane et al.’ 86 warm	$W=3.301\times 10^{-6}U_{10}^{3.479}$	
7	Bortkovskii [36]	Bortkovshii 87 cold	$W=0.189U_{10}^{}-1.28$	As a percentage
8	Bortkovskii [36]	Bortkovshii 87 moder	$W=1.71\times 10^{-5}U_{10}^{4.43}$	A percentage
9	Bortkovskii [36]	Bortkovshii 87 warm	$W=6.78\times 10^{-3}U_{10}^{2.76}$	As a percentage
10	Wu [37]	Wu 88	$W=1.7\times 10^{-6}U_{10}^{3.75}$	
11	Asher and Wanninkhof [25]	Asher and Wanninkhof 98	$W=2.56\times 10^{-6}{\left(U_{10}-1.77\right)}^{3}$	
12	Hanson and Phillips [38]	Hanson and Phillips 99	$W=3.66\times 10^{-9}U_{10}^{5.16}$	
13	Reising et al. [39]	Reising et al. 02	$W=3.5\times 10^{-6}{\left(U_{10}-0.6\right)}^{3}$	
14	Stramska and Petelski [40]	Stram & Petel. 03 all W	$W=4.18\times 10^{-5}{\left(U_{10}-4.93\right)}^{3}$	All W measured
15	Stramska and Petelski [40]	Stram & Petel. 03 de	$W=5.0\times 10^{-5}{\left(U_{10}-4.47\right)}^{3}$	Developed sea
16	Stramska and Petelski [40]	Stram & Petel. 03 unde	$W=8.75\times 10^{-5}{\left(U_{10}-6.33\right)}^{3}$	Undeveloped sea
17	Villarino et al. [41]	Villarino et al. 03 stable	$W=2.32\times 10^{-6}U_{10}^{3.4988}$	Stable conditions
18	Villarino et al. [41]	Villarino et al. 03 unstable	$W=0.43\times 10^{-6}U_{10}^{3.6824}$	Unstable conditions
19	Lafon et al. [42]	Lafon et al. 04	$W=1.51\times 10^{-4}U_{10}^{3.65}$	As a percentage, U > 5 m/s
20	Goddijn et al. [43]	Goddijn-Murphy et al. 11	$W=11.50\times 10^{-5}U_{10}^{1.86}$	
21	Salisbury et al. [20]	Salisbury et al. 13 F10	$W=4.6\times 10^{-5}U_{10}^{2.26}$	10 GHz, horizontal polarization
22	Salisbury et al. [20]	Salisbury et al. 13 F37	$W=39.7\times 10^{-5}U_{10}^{1.59}$	37 GHz, horizontal polarization

**Table 2:** Data source, grid resolution, variables used, and data access.

Model/Sensor Access	Variable	Resolution	Variable Used
Windsat (Coriolis)	Brightness temperature T_B_ (K)	0.5° × 0.5°	W(*T_B_*) algorithm
Naval Research Laboratory			
SSM/I (F13) Remote Sensing Systems ^1^	Water vapor Cloud liquid water	0.25° × 0.25°	W(*T_B_*) algorithm
SeaWinds (QuikSCAT)	Wind speed U_10_ (m s^−1^)	0.25° × 0.25°	W(*T_B_*) algorithm
PODAAC/JPL ^2^	Wind direction U_dir_ (°)		Whitecap coverage expression
			MASNUM Wave Model
GDAS/NCEP ^3^	U_10_, U_dir_, SST (°C)	1° × 1°	W(T_B_) algorithm

^1^. www.remss.com. ^2^. Physical Oceanography Distributed Active Archive Center at the NASA Jet Propulsion Laboratory [54]. ^3^. Global Data Assimilation System, National Centers for Environmental Prediction [55].

**Table 3:** Combinations of *θ* and *ρ*. The first row are the values for *θ* and the first column are the values for *ρ*.

	*θ*	8	8.6	9.2	9.8	10.4	11
*ρ*							
**0.53**		1	8	15	22	29	36
**0.54**		2	9	16	23	30	37
**0.55**		3	10	17	24	31	38
**0.56**		4	11	18	25	32	39
**0.57**		5	12	19	26	33	40
**0.58**		6	13	20	27	34	41
**0.59**		7	14	21	28	35	42

**Table 4:** Correlation coefficient (*r*) between satellite measurements and model results. The correlation coefficient r is related to the covariance of the whitecap coverage. The first row are the values for *θ* while the first column are the values for *ρ*. The significant level is 0.05. The critical value is 0.062.

		*θ*	8	8.6	9.2	9.8	10.4	11
	r							
*ρ*								
**0.53**			0.6267	0.6267	0.6265	0.6262	0.6262	0.6262
**0.54**			0.6059	0.6059	0.6059	0.6059	0.6059	0.6068
**0.55**			0.5835	0.5835	0.5829	0.5821	0.5821	0.5820
**0.56**			0.5558	0.5552	0.5546	0.5536	0.5536	0.5530
**0.57**			0.5250	0.5250	0.5250	0.5249	0.5249	0.5249
**0.58**			0.4977	0.4977	0.4977	0.4979	0.4982	0.4982
**0.59**			0.4717	0.4717	0.4717	0.4717	0.4717	0.4710

**Table 5:** Maximum and mean whitecap coverage over the western part (135° W–100° W, 44° S–56° S).

	Max (%)	Mean (%)
Model result	2.966	2.227
Satellite result	3.166	2.201

**Table 6:** Maximum and mean whitecap coverage over the eastern part (83° E–135° E, 44° S–56° S).

	Max (%)	Mean (%)
Model result	3.428	2.078
Satellite result	3.076	2.137

**Table 7:** Wind speed empirical relationships.

Reference	Empirical Relationship
Monahan et al. [58]	*W* = 4.5 × 10^−^^6^ *U*^3.31^
Salisbury et al. [20]	*W*= 3.97 × 10^−2^ × *U*^1.59^
Stramska M. and Petelski T. [40]	*W* = 4.18 × 10^−5^(*U* − 4.93)^3^
Wu [37]	*W* = 1.7 × 10^−6^ *U*^3.75^

## References

[1] Romero L., Lenain L., Melville W.K. (2017). Observations of Surface-Wave-Current Interaction. J. Phys. Oceanogr..

[2] Phillips O.M., Weyl P.K. (1977). The Dynamics of the Upper Ocean.

[3] Zhang S., Cao R., Xie L. (2012). Energy dissipation through wind-generated breaking waves. Chin. J. Oceanol. Limnol..

[4] He H.L., Song J.B. (2014). Determining the onset and strength of unforced wave breaking in a numerical wave tank. China Ocean Eng..

[5] Cardone V.J. (1970). Specification of the Wind Distribution in the Marine Boundary Layer for Wave Forecasting. Ph.D. Thesis.

[6] Monahan E.C., O’Muircheartaigh I.G. (1986). Whitecaps and the passive remote sensing of the ocean surface. Int. J. Remote Sens..

[7] Kraan G., Oost W.A., Janssen P.A.E.M. (1996). Wave Energy Dissipation by Whitecaps. J. Atmos. Ocean. Technol..

[8] Cavaleri L., Alves J.H., Ardhuin F., Babanin A., Banner M., Belibassakis K., Benoit M., Donelan M., Groeneweg J., Herbers T.H. (2007). Wave modelling—The state of the art. Prog. Oceanogr..

[9] Thomson J., Gemmrich J.R., Jessup A.T. (2009). Energy dissipation and the spectral distribution of whitecaps. Geophys. Res. Lett..

[10] Lamarre E., Melville W.K. (1991). Air entrainment and dissipation in breaking waves. Nature.

[11] Melville W.K., Loewen M.R., Lamarre E., Banner M.L., Grimshaw R.H.J. (1992). Sound Production and Air Entrainment by Breaking Waves: A Review of Recent Laboratory Experiments. Breaking Waves.

[12] Zhao D.L. (2012). Progress in sea spay and its effects on air-sea interaction. Adv. Earth Sci..

[13] Monahan E.C., Bryan R.K. (1988). Whitecap Coverage as a Remotely Monitorable Indication of the Rate of Bobbie Injection into the Oceanic Mixed Layer. Sea Surface Sound.

[14] Monahan E.C., Kerman B. (1990). Occurrence and evolution of acoustically relevant subsurface bubble plumes and their associated, remotely monitoriable, surface whitecaps. Natural Physical Sources of Underwater Sound.

[15] Potter H., Smith G.B., Snow C.M., Dowgiallo D.J., Bobak J.P., Anguelova M.D. (2016). Whitecap lifetime stages from infrared imagery with implications for microwave radiometric measurements of whitecap fraction. J. Geophys. Res..

[16] Randolph K., Dierssen H.M., Cifuentes-Lorenzen A., Balch W.M., Monahan E.C., Zappa C.J., Drapeau D.T., Bowler B. (2016). Novel methods for optically measuring whitecaps under natural wave breaking conditions in the Southern Ocean. J. Atmos. Ocean. Technol..

[17] Callaghan A., de Leeuw G., Cohen L., O’Dowd C.D. (2008). Relationship of oceanic whitecap coverage to wind speed and wind history. Geophys. Res. Lett..

[18] Dyachenko S., Newell A.C. (2016). Whitecapping. Stud. Appl. Math..

[19] Anguelova M.D., Webster F. (2006). Whitecap coverage from satellite measurements: A first step toward modeling the variability of oceanic whitecaps. J. Geophys. Res..

[20] Salisbury D.J., Anguelova M.D., Brooks I.M. (2013). On the variability of whitecap fraction using satellite-based observations. J. Geophys. Res..

[21] Gordon H.R. (1997). Atmospheric correction of ocean color imagery in the Earth Observing System era. J. Geophys. Res..

[22] Frouin R., Iacobellis S.F., Deschamps P.Y. (2001). Influence of oceanic whitecaps on the Global Radiation Budget. Geophys. Res. Lett..

[23] Monahan E.C., Spillane M.C., Brutsaert W., Jirka G.H. (1984). The Role of Oceanic Whitecaps in Air-Sea Gas Exchange. Gas Transfer at Water Surfaces.

[24] Woolf D.K., Liss P.S., Duce R.A. (1997). Bubbles and their role in gas exchange. The Sea Surface and Global Change.

[25] Asher W.E., Wanninkhof R. (1998). The effect of bubble-mediated gas transfer on purposeful dual-gaseous tracer experiments. J. Geophys. Res..

[26] Woolf D.K., Leifer I.S., Nightingale P.D., Rhee T.S., Bowyer P., Caulliez G., De Leeuw G., Larsen S.E., Liddicoat M., Baker J. (2007). Modelling of bubble-mediated gas transfer: Fundamental principles and a laboratory test. J. Mar. Syst..

[27] Woolf D.K. (2005). Parametrization of gas transfer velocities and sea-state-dependent wave breaking. Tellus.

[28] Guan C.L., Sun J. (2004). Similarities of some wind input and dissipation source terms. China Ocean Eng..

[29] Guan C.L., Hu W., Sun J., Li R. (2007). The whitecap coverage model from breaking dissipation parametrizations of wind waves. J. Geophys. Res..

[30] Yuan Y., Han L., Hua F., Zhang S., Qiao F., Yang Y., Xia C. (2009). The statistical theory of breaking entrainment depth and surface whitecap coverage of real sea waves. J. Phys. Oceanogr..

[31] Anguelova M.D., Hwang P.A. (2015). Using Energy Dissipation Rate to Obtain Active Whitecap Fraction. J. Phys. Oceanogr..

[32] Wang H., Yang Y., Sun B., Shi Y. (2017). Improvements to the statistical theoretical model for wave breaking based on the ratio of breaking wave kinetic and potential energy. Sci. China Earth Sci..

[33] Monahan E.C. (1971). Oceanic whitecaps. J. Phys. Oceanogr..

[34] Monahan E.C., Muircheartaigh I. (1980). Optimal Power-Law Description of Oceanic Whitecap Coverage Dependence on Wind Speed. J. Phys. Oceanogr..

[35] Spillane M.C., Monahan E.C., Bowyer P.A., Doyle D.M., Stabeno P.J., Monahan E.C., Niocaill G.M. (1986). Whitecaps and Global Fluxes. Oceanic Whitecaps.

[36] Bortkovskiĭ R.S., Monahan E.C. (1987). AirSea Exchange of Heat and Moisture during Storms. Atmos. Sci. Libr..

[37] Wu J. (1988). Variations of Whitecap Coverage with Wind Stress and Water Temperature. J. Phys. Oceanogr..

[38] Hanson J.L., Phillips O.M. (1999). Wind Sea Growth and Dissipation in the Open Ocean. J. Phys. Oceanogr..

[39] Reising S.C., Asher W.E., Rose L.A., Aziz M.A. Passive Polarimetric Remote Sensing of the Ocean Surface: The Effects of Surface Roughness and Whitecaps. Presented at the International Union of Radio Science.

[40] Stramska M., Petelski T. (2003). Observations of oceanic whitecaps in the north polar waters of the Atlantic. J. Geophys. Res..

[41] Villarino R., Camps A., Vall-Ilossera M., Miranda J., Arenas J. Sea foam effects on the brightness temperature at L-band. Proceedings of the Geoscience and Remote Sensing Symposium, IGARSS ′03.

[42] Lafon C., Piazzola J., Forget P., Le Calve O., Despiau S. (2004). Analysis of the Variations of the Whitecap Fraction as Measured in a Coastal Zone. Bound-Lay. Meteorol..

[43] Goddijnmurphy L., Woolf D.K., Callaghan A.H. (2011). Parameterizations and Algorithms for Oceanic Whitecap Coverage. J. Phys. Oceanogr..

[44] Sugihara Y., Tsumori H., Ohga T., Yoshioka H., Serizawa S. (2007). Variation of whitecap coverage with wave-field conditions. J. Mar. Syst..

[45] Zhao D., Toba Y. (2001). Dependence of Whitecap Coverage on Wind and Wind-Wave Properties. J. Oceanogr..

[46] Kinsman B. (1965). Wind waves: Their generation and propagation on the ocean surface. Fungal Biol.-UK.

[47] Jones I.S.F., Toba Y. (2001). Wind Stress over the Ocean.

[48] Yang Y.Z., Qiao F.L., Pan Z.D. (2000). Wave Assimilation and Numerical Prediction. Chin. J. Oceanol. Limnol..

[49] Yang Y., Ji Y., Yuan Y. (2003). The nonlinear interaction process in the wave assimilation model and its experiments. Chin. J. Oceanol. Limnol..

[50] Jiang X., Wang D., Gao D., Zhang T. (2016). Experiments on exactly computing non-linear energy transfer rate in MASNUM-WAM. Chin. J. Oceanol. Limnol..

[51] Yang Y.Z., Qiao F.L., Zhao W., Teng Y., Yuan Y. (2005). MASNUM ocean wave numerical model in spherical coordinates and its application. Acta Oceanol. Sin..

[52] Anguelova M.D., Bobak J.P., Asher W.E., Dowgiallo D.J., Moat B.I., Pascal R.W., Yelland M.J. Validation of satellite-based estimates of whitecap coverage: Approaches and initial results. Proceedings of the 16th Air-Sea Interaction Conference, AMS.

[53] Anguelova M.D., Gaiser P.W. (2013). Microwave emissivity of sea-foam layers with vertical profile of dielectric properties. Remote Sens. Environ..

[54] Data. https://aquarius.oceansciences.org/cgi/data.cgi.

[55] EMC: Data Assimilation Team. Grid-Point Statistical Interpolation (GSI). http://www.emc.ncep.noaa.gov/gmb/gdas/.

[56] Nordberg W., Conaway J., Ross D.B., Wilheit T. (1971). Measurements of Microwave Emission from a Foam-Covered, Wind-Driven Sea. J. Atmos. Sci..

[57] Ross D.B., Cardone V. (1974). Observations of oceanic whitecaps and their relation to remote measurements of surface wind Speed. J. Geophys. Res..

[58] Monahan E.C., Fairall C.W., Davidson K.L., Boyle P.J. (1983). Observed interrelation between 10 m winds, ocean whitecaps and marine aerosols. Q. J. R. Meteorol. Soc..

[59] Scanlon B., Breivik Ø., Bidlot J.R., Janssen P.A., Callaghan A.H., Ward B. (2015). Modeling Whitecap Fraction with a Wave Model. J. Phys. Oceanogr..

